# Scraps

**Published:** 1896-09

**Authors:** 


### SCRAPS

PICKED UP BY THE ASSISTANT-EDITOR.

**Septic Surgery.**—We have left unwashed those things which we ought to have washed, and we have handled those things which we ought not to have handled, and there is no health in us.—*Medical Record.*

**A Scotch Pharmacopœia.**--A stranger came to Jedburgh one day,—or, as the natives prefer to call it, "Jeddart." He looked somewhat of a valetudinarian, and he asked one of the inhabitants to direct him to the chemist's shop. "The what, sir?" "The chemist's shop." "Ay, an' whit kin' o' a shop's that na?" "Why, the place where you can buy medicine!" "Eh, sir; we 've nae sic shop as that in Jeddart!" "No? What do you do, then, when any one falls ill? Do you take no medicine?" "'Deed, no! We've jist whiskey for the folk an' tar for the sheep, an' hat' s a' the feesick we deal in!"

**Clinical Records** (16).—An American paper is responsible for the following: "What can I do for you, miss?" inquired the clerk in a drugstore. The blushing young woman glanced about her in embarrassment, and then replied: "I want some castor oil." "All right; in just a moment." The clerk moved around behind the counter for a moment, and then went to the soda-fountain. "Do you like soda?" he asked. "Oh, yes, indeed." "What flavouring do you prefer?" "Pine-apple, please." The clerk drew a glass of the fizz, and the young lady drank it. Then the clerk sat down on a stool and commenced to chat with her. She was apparently annoyed, but replied courteously to all his remarks. Finally she said: "If you'll give me the castor oil, I'll go." "Why, you took it in that glass of soda." "Took it in the soda! I didn't want to take it. It was for my little brother." And the clerk wondered why she was indignant.

**Serumtherapy.**—Dr. Granville P. Conn, pleading for "Conservatism in Medicine and Surgery" before the New Hampshire Medical Society, made some caustic remarks upon the extravagant pretensions put forth on behalf of many modern preparations, and quoted the following lines from the pen of a doctor:

An extract of muscles for rheumatic pains.
A grey-matter extract to nourish our brains.
An extract of teeth for a man that can't chaw.
A maxillary extract to cure lock-jaw.
An extract of ocean to cure *mal de mer.*
A hirsutic extract for those without hair.
A duodenal extract to serve in good turn
In healing the ulcers that follow a burn.
An extract made out of a whole population,
To rescue some housewife from sterilisation.

**Medical Philology** (XIX.).—In the *Promptorium* occurs the following: "CODE, sowters wax. *Coresina.*" Variant readings are "coode" and "ceresina." Neither Mr. Way, the editor of the *Promptorium*, nor the *New English Dictionary* throws any light on this now obsolete Middle English word for cobblers' wax. It has a medical interest, for Mr. Way gives this note: "Among numerous substances, resin, grease, and herbs, mentioned in the curious directions for making a good 'entreet,' or plaster to heal wounds, occurs 'Spaynisch code.' Sloan. MS. 100, f. 17." Plasters for various purposes were much in favour with some of our forefathers. Many interesting instances can be seen in *Select Observations on English Bodies of Eminent Persons in Desperate Diseases*, written by Shakspere's son-in-law, Dr. John Hall. The 1683 edition of this work has been lately added to the Bristol Medical Library. In the *Journal* for September, 1891, I gave some notes on Dr. Hall's therapeutics.

**The New "Photography."**—Apropos of the wonderful penetrating powers of the cathodic rays, a young lady, at a recent dinner party in New York, ventured the remark that she understood that these new cathartic rays could go through anything.—*The Journal of the American Medical Association.*

The teller of sensational stories gets along faster with the methods of the X rays than the scientific student does. In a story in the *Strand Magazine* for July, among other inconsistencies occurs an account of the discovery of a diamond set in gold which an Indian servant had stolen from his master and then swallowed in order to prevent detection. "A Man of Science" (!) says:

"I arranged him in such a position that the rays should pass through his body. I turned off the light in the room—my electrical battery worked well, the rays played admirably in the vacuum tube. I removed the cap from the camera, and after an exposure of from seven to ten minutes, felt certain that I had taken a careful photograph."

**Sanitary Science.**—In the *Journal* for June, 1895, I quoted some answers which had been given at a test-examination for Sanitary Inspectors. Here are a few more taken from an article by Mr. H. Percy Boulnois in the *Sanitary Record.* Asked to define a "Common Lodging House," one candidate replied: "A common lodging-house is a place where those of the public who have not the advantage of a home of their own may pass the night;" and another still more vaguely said: "A common lodging-house is a place where a person can get a night's lodging or more;" what the *more* refers to we must leave to those who have enjoyed a night's lodging in such an abode. It is curious to note how "mixed" some of the candidates get when speaking of the separation of the sexes in common lodging-houses. One of them simply said: "A house used by strangers promiscuously for purposes of habitation;" whilst another defined it as "A house where people, not of the same family, may sleep together under the control of the Local Authority;" whilst a third said: "On no account must opposite sexes be allowed to sleep in the same room in different beds."

**Medical Libraries.**—Some publishers may be so short-sighted as to think that every book that they put into a medical college library will diminish many-fold that book's sale. This can only be the case when the book is unworthy. The college library, in conjunction with the study of medical literature in the college course, would increase the sale of the best medical literature, and, it is to be hoped, would diminish in some degree the sale of books of low degree now being so persistently exploited upon the medical profession. The systems and text-books of the commercial doctors and cheap publishers are debauching now the taste of the profession. Some radical means must be taken to counteract this influence. The study of medical literature in medical schools is a promising educational advance, and a powerful adjunct to a better professional life. No other means of study gives so much interest to medical and surgical investigation. The broad outlook on life, and on professional life especially, that every young medical man should possess, is best inculcated by a study of the inheritance transmitted through our literature.—*Medical News.*

**Obstetrics Home and Foreign** (2).—There were some ceremonies anciently used when the lady took her chamber. It is stated that when the Queen of Henry VII. took her chamber in order to her delivery, "the Erles of Shrewsbury and of Kente hyld the towelles, whan the quene toke her rightes; and the torches were holden by knightes. When she was comen into hir great chambre, she stode undre hir cloth of estate; then there was ordeyned a voide of espices and swet wyne; that doone, my lorde, the quenes chamberlain, in very goode wordes desired, in the Quene's name, the pepul there present to pray God to *sende hir the good oure;* and so she departed to her inner chambre." Strutt, iii. 157, from a MS. in the Cotton Library.—Brand's *Popular Antiquities*, ed. Bohn, 1849, vol. II, p. 66.

The Indian squaw is wont to steal off into the woods for her confinement. Alone or accompanied by a female relative or friend she leaves the village, as she feels the approach of labour, to seek some retired spot; upon the banks of a stream is the favourite place the world over, the vicinity of water, moving water, if possible, is sought, so that the young mother can bathe herself and her child, and return to the village cleansed and purified when all is over. In winter, a temporary shelter is erected in the vicinity of the family lodge by those who make the solitude of the forest their lying-in chamber in milder weather. Some of the Sioux tribes, the Blackfeet and the Uncapapas, are in the habit of arranging a separate lodge, generally a temporary one, for the occasion; as also do the Klamaths, the Utes, and others. The Comanches construct a shelter for parturient women a short distance outside of the camp and in the rear of the patient's family lodge.—Engelmann's *Labor among Primitive Peoples*, 1882, pp. 126-7.

